# Unlocking Solid Polymer Electrolytes: Advancing Materials through Characterization-Driven Insights

**DOI:** 10.1021/jacsau.5c00442

**Published:** 2025-07-07

**Authors:** Alberto Alvarez-Fernandez, Guiomar Hernández, Jon Maiz

**Affiliations:** † 202635 Centro de Física de Materiales (CFM-MPC), CSIC-UPV/EHU, 20018 Donostia - San Sebastián, Spain; ‡ Department of Chemistry − Ångström Laboratory, 8097 Uppsala University, Box 538, SE-751 21 Uppsala, Sweden; § IKERBASQUE − Basque Foundation for Science, Plaza Euskadi 5, 48009 Bilbao, Spain

**Keywords:** Solid Polymer Electrolytes, Batteries, Scattering, Spectroscopy, Ionic Conductivity

## Abstract

Solid polymer electrolytes (SPEs) hold great promise for next-generation battery technologies due to their inherent safety and mechanical stability. However, widely used poly­(ethylene oxide) (PEO)-based electrolytes face significant challenges, including high crystallinity, low ionic conductivity at ambient temperatures, and a narrow electrochemical stability window. Overcoming these limitations requires the development of novel polymer matrices alongside the refinement of advanced characterization methods that capture the fundamental dynamics of ion transport and polymer segmental mobility. In this Perspective, we review recent advancements in SPE design, focusing on innovative materials such as polytetrahydrofuran (PTHF) or poly­(trimethylene carbonate) (PTMC) as well as solid composite electrolytes. We also examine alternative synthetic strategies, including copolymerization, blending, and cross-linking, which aim to reduce crystallinity and enhance ion conduction. Importantly, we emphasize the urgent need for comprehensive experimental and computational characterization techniques. Progress in small-angle X-ray and neutron scattering, quasielastic neutron scattering, and in situ spectroscopy has provided critical insights into the complex interactions between ions and polymer chains. By integrating innovations in materials synthesis with state-of-the-art characterization approaches, this work outlines a forward-looking roadmap for the rational design of SPEs that can meet the demanding requirements of next-generation energy storage systems.

## Graphical abstract

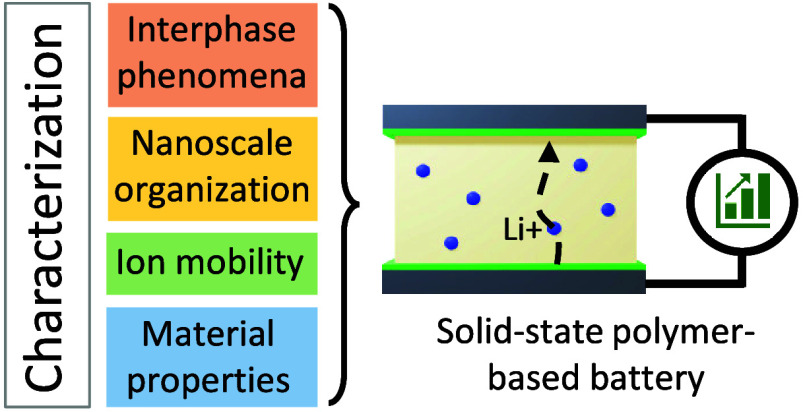

## 1 Introduction

Solid polymer electrolytes (SPEs) have become a key focus in the development of lithium-based batteries, driven by the need for improved safety and higher energy density.^1,2^ By replacing traditional liquid electrolytes, SPEs eliminate the risk of leakage and combustion, while providing a flexible and mechanically robust medium that can better accommodate the high-voltage cathodes and lithium–metal anodes required for modern battery systems.^3–7^ These features not only address safety concerns but also create opportunities for designing batteries that can operate across a wider range of temperatures and applications.^8,9^

Poly­(ethylene oxide) (PEO) remains the benchmark polymer for SPEs due to its intrinsic ability to solvate lithium ions (Li^+^) via its ether oxygen groups.^10,11^ In PEO-based systems, (Li^+^) are predominantly coordinated by the oxygen atoms along the polymer backbone, forming a helical structure that facilitates ion transport within the amorphous regions.^12,13^ However, the semicrystalline nature of PEO at ambient temperature restricts chain mobility, leading to low ionic conductivity and a narrow electrochemical window (typically below 3.8 V vs Li/Li^+^).^14–16^ These limitations hinder fast charge/discharge rates and compromise interfacial stability with high-voltage cathodes.^17,18^

To address these challenges, PEO’s well-understood coordination chemistry has served as a foundation for developing advanced electrolyte formulations, where modifications such as copolymerization and additive incorporation aim to mitigate its inherent drawbacks.^19^ For example, additives such as silica nanoparticles or other inorganic species have proven effective in modifying the solvation environment in PEO-based systems, enhancing lithium salts dissociation and improving polymer segmental motion.^20^ Complementary to these additive strategies, copolymerization has been effectively used to tailor the solvation environment. For instance, synthesizing single-ion conducting diblock copolymers by combining PEO with sulfonylimide-based segments induces microphase separation, leading to well-defined ion channels that significantly enhance Li^+^ mobility and overall electrolyte performance.^21,22^ Moreover, blending approaches, such as mixing PEO with polymers like poly­(acrylonitrile) (PAN) or poly­(methyl methacrylate) (PMMA), disrupt the crystalline order of PEO,^23–25^ thereby increasing the amorphous fraction and further boosting ionic conductivity.

Nonetheless, challenges persist with PEO-based electrolytes, driving growing interest in alternative SPEs.^26^ Polymer systems such as poly­(tetrahydrofuran) (PTHF),^27^ or poly­(trimethylene carbonate) (PTMC)^28^ have gained attention for their naturally lower crystallinity and higher amorphous content, which enhances chain mobility and (Li^+^) transport.

In this pursuit, simulation and predictive analysis tools have been indispensable. For instance, molecular dynamics (MD) simulations of PTHF reveal a looser Li^+^ coordination environment, where fewer oxygen atoms participate, leading to lower desolvation energy barriers and enhanced ion mobility.^29^ Similar computational studies on PTMC indicate that their intrinsically amorphous structures enable more efficient Li^+^ diffusion,^30^ guiding experimental efforts and facilitating the development of a new library of SPEs with improved performance.

Furthermore, researchers have recognized that advancements in SPE performance extend beyond the electrolyte material itself. Modifications of electrode surfaces have become an integral strategy for enhancing overall battery function.^31,32^ Techniques such as applying functional coatings or incorporating tailored interlayers on electrode surfaces help reduce interfacial impedance, stabilize the electrode–electrolyte interface, and suppress lithium dendrite formation.^33,34^ These surface modifications, in conjunction with advanced SPE designs, contribute to batteries with longer cycle life and higher efficiency.

Despite advances in synthesis and computational tools, characterization techniques have lagged behind. Besides glass transition temperature (*T*_g_) and ionic conductivity measurements, other common experimental characterization efforts have focused on structural and chemical aspects, utilizing tools such as atomic force microscopy (AFM),^35,36^ X-ray scattering techniques,^21^ and Raman and infrared spectroscopies.^37,38^ While these methods provide valuable insights into the segmental and ionic mobility as well as morphological changes and local chemical environments of SPEs, they fall short in capturing dynamic events such as ion migration, ion coordination strength, and ion transport mechanism.

Time-resolved approaches, such as broadband dielectric spectroscopy (BDS), quasielastic neutron scattering (QENS), or neutron spin echo (NSE) show promise in bridging this gap but are not yet widely implemented.^39–41^ Additionally, the integration of machine learning and artificial intelligence with experimental and computational tools holds the potential to offer a more comprehensive understanding of SPE dynamics, ultimately guiding the development of electrolytes with enhanced performance.^42^

We are at a critical juncture in the evolution of SPEs, as material chemists, engineers, and physicists collaborate to address long-standing challenges. Advances in synthesis, dynamic characterization, and computational tools now work in unison, enabling researchers to take a multifaceted approach to improving SPE performance. This interdisciplinary effort is paving the way for tailoring ion transport, enhancing interfacial stability, and ultimately achieving the high ionic conductivity and robust electrochemical properties required for next-generation lithium-based batteries and beyond (Figure 1).

**Figure 1:**
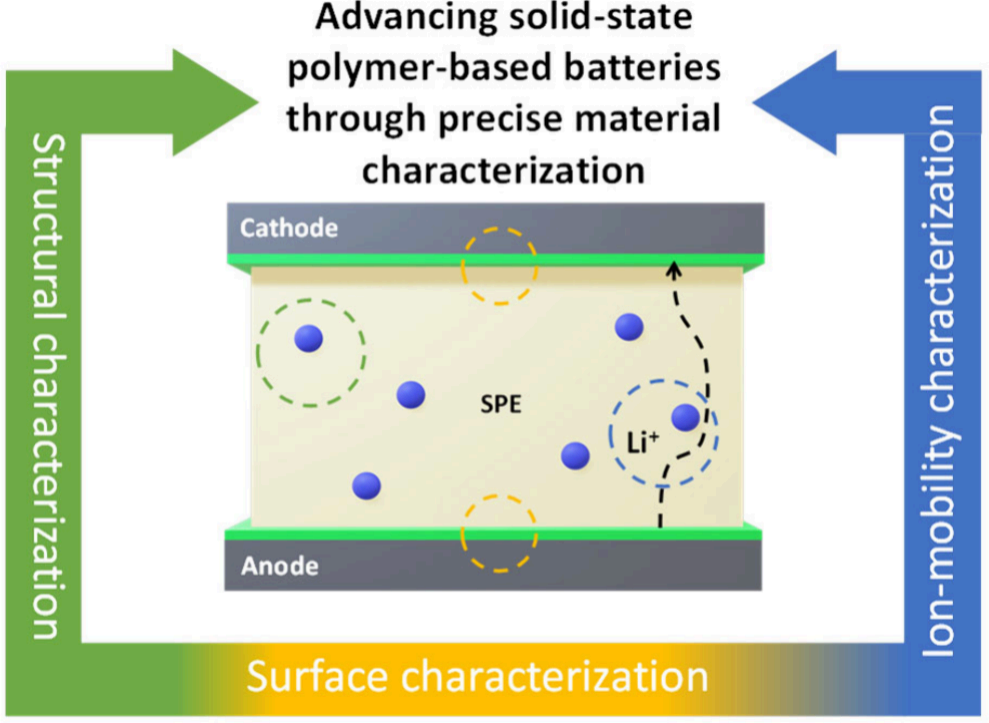
Schematic framework of this Perspective: from the synthesis of alternative materials to their structural, surface, and ion-mobility characterizations.

This Perspective aims to contribute to this progress by giving a critical overview of the recent developments in terms of characterization techniques and polymer design, including the benefits and limitations. Furthermore, a roadmap is proposed for accelerating the advancement of novel time-resolved characterization techniques that provide additional insights into the materials’ properties, computational methods, and novel polymer architectures toward next-generation SPEs.

## 2 SPEs: Why Now?

Across sectors, from electric vehicles and wearables to grid-level storage and aerospace, manufacturers are looking for ways to raise energy density without compromising safety. One solution is to remove the fire-prone electrolytes used in today’s (Li^+^) cells. Early industrial efforts tried to solve the liquid-electrolyte problem with gels and “semi-solid” pastes. These formulations reduce leakage but still carry organic solvent and thus remain flamable, limiting their appeal for many targeted applications. Another solution is fully solid-state electrolytes. Inorganic oxides and sulfides deliver room-temperature conductivities rivalling liquids, yet their brittleness, sensitivity to moisture, and need for high-temperature sintering have slowed the jump from coin cell to large scale implementation of these electrolytes. It is this manufacturing gap, rather than conductivity alone, that has opened the door for SPEs. Polymers can be slit-coated and laminated on existing roll-to-roll lines, conform naturally to lithium–metal anode’s surface, and do not require tonne-scale stack pressure, making them an immediately compatible upgrade for today’s solid-state battery manufacturers.

SPE-based technology has already made its way to market in some cases. The most well-known example is Blue Solutions, a subsidiary of the Bolloré Group. The company began developing its lithium-metal polymer (LMP) battery technology in the 1990s,^43^ with industrialization launched in 2001 through its subsidiary Batscap. Initially, the cathode was based on vanadium oxide (VOx), conductive additives, and a PEO-based polymer electrolyte, with lithium-metal as the anode. The polymer served dual roles, as an ionic conductor and a binder, ensuring close contact with the active material and enhancing compatibility with the solid-state electrolyte layer. The cathode mixture was extruded onto an aluminum current collector, while the bulk electrolyte, comprising polymer and salt, was simultaneously extruded and deposited onto the cathode. Blue Solutions’ batteries are commercially deployed under the Blue Systems brand in electric vehicles and buses. The Bluecar uses a 30 kWh LMP battery with a volume of 300 L and a weight of 300 kg. It delivers an urban range of around 250 km, with internal operating temperatures of 60–80 °C and external operating limits from −20 to 160 °C. The Bluebus series includes a 6-m model with a range of up to 280 km and a 12-m model reaching up to 380 km. Blue Solutions has announced a €2.2 billion investment in a 25 GWh polymer-electrolyte gigafactory to support next-generation electric vehicle cells,^44^ showing the great level of maturity achieved by their technology.

Other alternative companies are also making important steps to bring SPEs to market. Blue Current is completing a 22,000 ft^2^ pilot line in Hayward, California, where it will dry-coat silicon-polymer solid-state cells for qualification runs in 2025.^45^ In North Carolina, Soelect has raised US$11 million in funding led by GM Ventures to scale its dendrite-resistant polymer electrolyte for large pouch formats.^46^ BasqueVolt (Spain) has raised an initial €10 million and earmarked more than €700 million for a 10 GWh plant due in 2027.^47^ Moreover, Nuvvon’s UL-certified solid-polymer pouch cells are cleared for global air-freight shipment, a milestone not yet matched by ceramic-based solid-state cells,^48^ and marking an important step in demonstrating the high level of safety of SPE-based batteries.

Despite these industrial strides, SPEs remain a vibrant research frontier. The center of gravity is shifting from “make-and-measure” materials work to integrated programs in which operando neutron and X-ray techniques, spectroscopy, and electrochemical testing run side-by-side, allowing researchers to watch morphology, segmental dynamics, and ion transport evolve in real time and to feed that insight straight back into molecular design.

## 3 Current Challenges in SPE Development: A Characterization Perspective

Translating promising SPEs formulations into reliable battery components is complicated not only by intrinsic material properties and interfacial issues but also by the limitations of current characterization techniques. A detailed evaluation from the characterization standpoint reveals several critical constraints that must be addressed to bridge the gap between laboratory research and practical applications.

### 3.1 Characterizing Polymer Material Properties

From the polymer perspective, the balance between a polymer’s thermal characteristics, such as its *T*_g_, and its molecular structure, notably its crystallinity, remains a significant challenge in SPE development. Highly crystalline domains provide structural stability, yet they restrict the segmental mobility essential for efficient Li^+^ migration. In practice, this means that excessive crystallinity can constrain ionic conductivity to levels below 10^–5^ S/cm at ambient temperatures, often necessitating the operation of devices at elevated temperatures (typically between 60 and 80 °C for PEO-based electrolytes) to achieve acceptable performance.^15^ Consequently, optimizing new matrices demands an accurate picture of their physico-thermal properties.

Conventional thermal analysis provides only a partial view.^49^ Differential scanning calorimetry (DSC) is routinely used to locate *T*_g_ and estimate the amorphous fraction, yet its accuracy is often affected by the sample’s thermal history. Thermogravimetric analysis (TGA) complements DSC by providing valuable data on thermal stability and degradation profiles, but because it reflects bulk properties, it may overlook minor degradation events that could influence long-term battery performance. X-ray diffraction (XRD) is used to measure the overall crystallinity of the polymer.^50^ However, its resolution is not high enough to detect small differences or localized stress-induced changes in lattice order that influence ion transport. Moreover, these techniques are normally conducted under controlled, steady conditions, far from the temperature changes and electrochemical stresses of working cells.

### 3.2 Challenges in Structural Characterization of SPEs

Beyond their thermal and conductive properties, understanding the microstructural organization of SPEs is crucial, as the arrangement and morphology of polymer chains directly dictate ion transport pathways.^51^ This challenge becomes even more pronounced with the advent of complex polymer chain topologies, such as random, block, and bottlebrush copolymers, whose phase behavior and microphase separation determine whether ion-conducting domains form a continuous network.^52,53^

One of the most used techniques for that is the AFM, which can deliver nanometre-scale surface topography and mechanical contrast; however, its analysis is generally confined to μm-scale surface area, and it is susceptible to artifacts arising from tip–sample interactions.^54^ Alternative microscopy techniques, such as scanning electron microscopy (SEM) and transmission electron microscopy (TEM), have demonstrated the ability to provide high-resolution surface as well as cross-sectional images of phase-segregated systems.^55,56^ However, these methods, particularly TEM, demand extensive sample preparation under high-vacuum conditions. This requirement not only risks altering the native morphology but also limits the feasibility of in situ or operando observations during battery operation, where dynamic changes in chain arrangement are crucial. Finally, small-angle X-ray and neutron scattering techniques (SAXS, SANS) have proved useful in providing bulk statistical information on domain sizes, shapes, and spatial distributions, revealing the average structural features and microphase separation phenomena that govern ion transport pathways.^57–60^ However, they are highly sensitive to factors such as sample inhomogeneity and contrast variations, particularly in systems with low electron or neutron scattering contrast, which can complicate data analysis and limit localized structural details. Additionally, the interpretation of scattering data often relies on complex modeling and fitting procedures that may oversimplify the inherent heterogeneity of the material.

### 3.3 Limited Understanding of Ion-Transport Mechanisms

Beyond characterizing the polymer matrix itself, it is equally important to understand how ions move through it for SPEs optimization. Existing models, including polymer segment migration, ion hopping, the Vogel–Fulcher–Tamman (VFT) model, Arrhenius behavior, and free volume theories, explain part of the picture. Still, none captures the full complexity of ionic conduction in SPEs. Thus, for example, in amorphous domains, ion transport is largely coupled with polymer segmental motions. Thus, ions migrate through sequential coordination and decoordination along the polymer backbone, a process that is generally well-described by VFT-type behavior. However, this model does not account for the existence of ion pairing and aggregation, phenomena that are common due to the relatively low dielectric constants of many host polymers. In contrast, in crystalline regions, ions may travel through well-defined channels via a hopping mechanism between vacancies, although this process is generally slower and less efficient compared to transport in amorphous phases. Moreover, the interplay between ion–polymer coordination and the dynamic rearrangement of polymer segments further complicates the picture. For example, strong coordination between Li^+^ and polar functional groups can facilitate salt dissociation, yet overly tight binding may impede ion mobility.

Characterization tools also reflect these ambiguities. Alternating current (AC) impedance spectroscopy remains the primary method for evaluating bulk conductivity in SPEs,^61^ however, real-world systems often deviate from ideal behavior; Nyquist plots frequently exhibit depressed arcs and inclined spikes that indicate a broad distribution of relaxation times arising from surface roughness, interfacial inhomogeneities, and microstructural complexities, making it difficult to fit the results with the methods previously mentioned. BDS extends the frequency window and, in principle, separates polymer relaxations from ionic motion.^39^ However, the extensive frequency range can result in overlapping relaxation processes, making it practically difficult to clearly separate the different contributions. Furthermore, electrode effects and sample geometry can also influence the measurements.

### 3.4 Interphase and Surface Characterization

A complete description of an SPE-based cell also requires insights into the ultrathin regions where the SPE is in contact with electrodes. Conventional tools reveal global morphology, thermal behaviors, and conductivity, but lack the spatial or temporal resolution to follow the nanometer-scale, rapidly evolving chemistry of the solid electrolyte interphase (SEI) on anodes or the cathode electrolyte interphase (CEI) on cathodes, and attempts to separate the components often alter the interphase.

New surface-sensitive techniques are beginning to fill this gap.^62^ Quantitative mechanical (QNM), Kelvin-probe (KPFM), and conductive (c-AFM) modes of AFM map variations in stiffness, surface potential, and local electronic leakage across SPE films, exposing spots where poor adhesion or stray currents could trigger failure.^35^ Cryogenic TEM allows researchers to visualize the interphase structure at near-atomic resolution under conditions that preserve the native state of the electrolyte.^63^ Time-of-flight secondary ion mass spectrometry (ToF-SIMS) further complements these approaches by providing detailed chemical profiles of the interphase, enabling the identification of functional groups, degradation products, and other chemical species that influence ion conduction.^64^ X-ray photoelectron spectroscopy (XPS) complements previous techniques by tracking shifts in core-level binding energies as the polymer or salt decomposes,^65^ clarifying how passivating interphases form a critical aspect in battery performance and lifespan.^66,67^ Most of these experiments are still carried out ex-situ or under static conditions, which may not fully capture the dynamic nature of the SEI, its formation, growth, and degradation under cycling conditions. Moving the same spatial resolution into an operando environment remains the next urgent step toward linking interphase evolution to ion transport and long-term battery performance.

## 4 Strategies for Next-Generation Solid Polymer Design

Recent advances in characterization techniques have provided a clearer picture of the structural and dynamic properties of SPEs. This improved understanding has led to new design strategies that adjust polymer architecture, manage interfaces, and incorporate specific additives. In this section, we discuss several approaches that build on these insights to improve ion transport and ensure long-term stability in SPEs.

### 4.1 From Structure to Performance: Engineering PEO-Based SPEs

As previously introduced, PEO is widely regarded as the gold standard for SPEs due to its excellent salt solvating properties and ion transport capabilities. However, its inherent crystallinity can restrict ion mobility and limit the performance and mechanical properties of the final device. Recent advances in characterization techniques, along with improvements in synthetic protocols, have offered new alternatives to mitigate PEO’s crystallinity and enhance its mechanical properties. This section highlights three key strategies: copolymerization, blending, and cross-linking.

#### 4.1.1 Copolymerization

Copolymerization has emerged as a versatile approach to tailor SPEs by integrating distinct polymer segments that simultaneously deliver high ionic conductivity and robust mechanical strength. Among the various chain architectures, e.g., random, alternating, gradient, and graft, block copolymers have attracted the most attention due to their ability to form well-defined, phase-separated morphologies.^68^ Engineering parameters such as the molecular weight (*M*_w_) and the volume fraction of each block enable the formation of diverse morphologies ranging from cylinders and lamellae to gyroids with controllable dimensions in the range of 10–100 nm. These structured domains create defined pathways that significantly enhance ion transport.

The development of more controlled synthetic approaches has allowed for unprecedented fine-tuning of block copolymer electrolytes, achieving precise control over the microphase-separated morphology.^69^ For instance, Butzelaar et al. demonstrated a novel RAFT polymerization strategy to synthesize polystyrene-*block*-poly­(ethylene oxide) (PS-*b*-PEO) block copolymers, successfully providing long-range ordered lamellar structures wherein ion-conducting PEO domains are seamlessly integrated with mechanically robust polystyrene regions.^70^ Ring opening polymerization (ROP) and other advanced synthetic approaches have also been recently applied in the synthesis of highly efficient SPEs.^71,72^

Alongside these synthetic innovations, breakthroughs in characterization techniques have underscored the potential of block copolymers as powerful platforms for systematically investigating transport phenomena. Beyond conventional ex situ methods, a suite of novel in situ and operando characterization platforms has emerged to probe the dynamic behavior of these materials under working conditions. For instance, in situ SAXS measurements have revealed that under polarization, block copolymer electrolytes can undergo reversible transitions between hexagonally packed cylindrical (HEX) and body-centered cubic (BCC) morphologies, directly affecting ion transport pathways.^73^ Alternatively, operando X-ray tomography has also proven invaluable for understanding how the electrolyte’s morphology influences battery performance, tracking the growth of lithium protrusions on metal anodes interfaced with a PS-*b*-PEO electrolyte.^74^ Their work established a clear correlation between the suppression of dendritic growth and the controlled phase structure of the block copolymer electrolyte. Complementary studies further illustrate the intricate coupling between morphology and transport. Galluzzo et al. used operando X-ray photon correlation spectroscopy (XPCS) to directly measure polymer velocities and cation transference number,^75^ revealing that field-induced morphological transitions critically influence ion transport and challenging conventional single-solvent approximations (Figure 2A).

**Figure 2:**
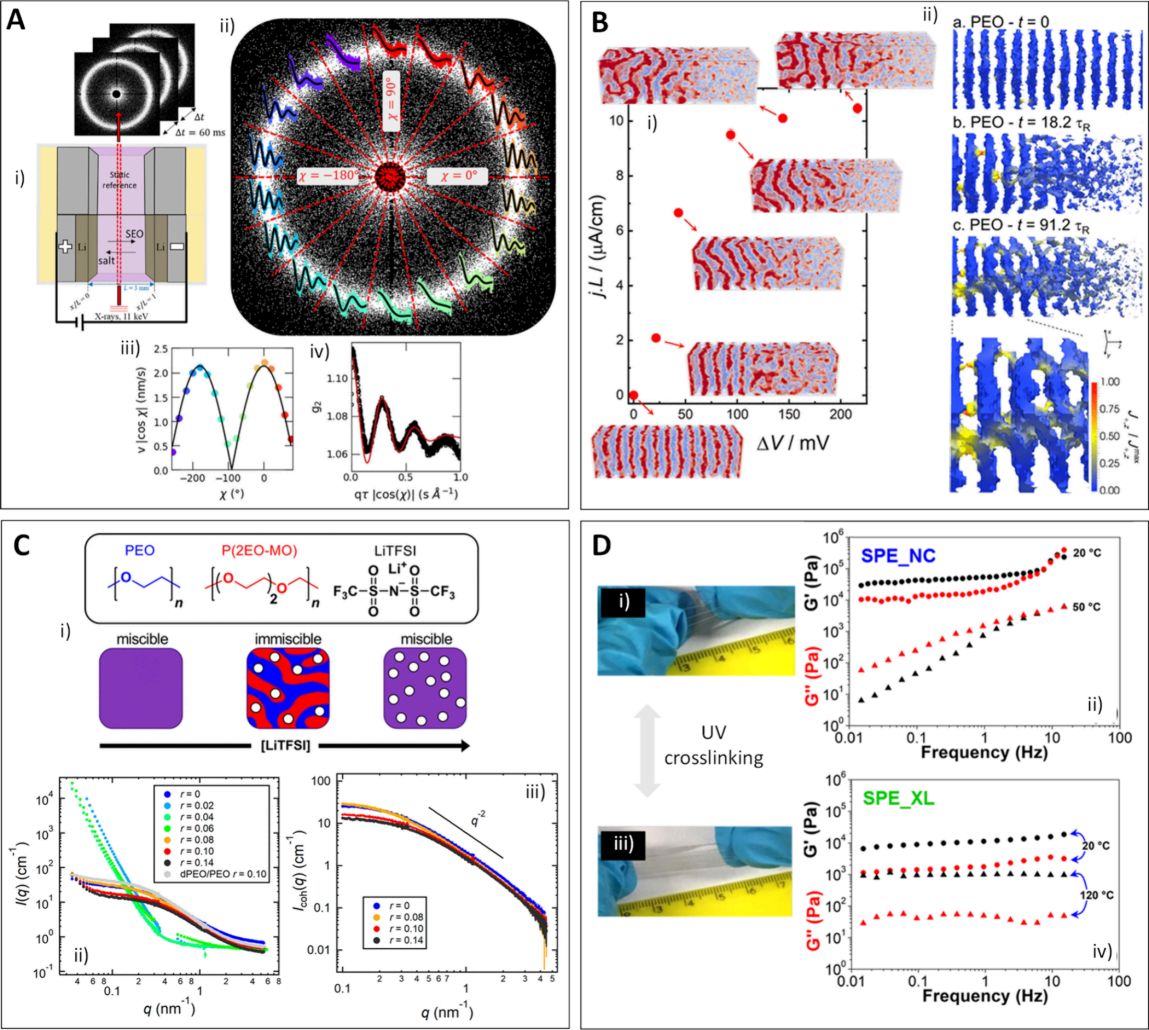
(A) (i) XPCS experimental setup. (ii) Representative SAXS pattern for one measurement obtained at *t* = 1.4 h during a 200-mV mm^–1^ polarization. (iii) Velocity, *v*|cos χ|, measured as a function of χ. (iv) Result of collapsing the autocorrelation functions along all χ values. Adapted from ref 75. CC BY-NC-ND 4.0. (B) (i) Steady-state current–length product, *jL*, as a function of the electric bias applied between the electrodes, Δ*V*. The insets show snapshots of the system. (ii) 3D images of the PEO component at different simulation times. Regions with Φ_PEO_ < 0.95 are transparent. The surface is colored according to the *z* component of the flux of Li^+^. Adapted from ref 76. CC BY 4.0. (C) (i) Schematic showing the blend strategy. (ii, iii) SANS intensity plots for (ii) the PEO/P­(2EOMO) blends at different compositions and (iii) the dPEO/P­(2EOMO)/LiTFSI blends at the miscible window. Adapted from ref 83. Copyright 2020 American Chemical Society. (D) Photographs of the polymer electrolytes (i) before and (iii) after UV exposition and (ii, iv) the corresponding rheology data. Adapted from ref 92. Copyright 2019 American Chemical Society.

An important simulation effort has been dedicated to understanding both the phase segregation behavior of SPEs and ion transport through nanostructured electrolytes. In this sense, it is especially interesting the work developed by Tagliazucchi et al. for providing molecular-level insights into how ionic currents drive morphological transitions, from lamellar to bicontinuous structures, in PS-*b*-PEO systems.^76^ This study elucidates how such transitions modulate ionic fluxes and conductivity under operando conditions (Figure 2B).

#### 4.1.2 Polymer Blending

Polymer blends as SPEs offer promising routes for enhancing battery performance.^77^ Merging crystalline and amorphous polymers enables a fine-tuned microstructure that harmonizes ion conduction with mechanical strength. Much of the research in this area has centered on blending PEO with homopolymers such as polyvinylidene fluoride (PVDF),^78^ polyacrylonitrile (PAN),^79^ thermoplastic polyurethane (TPU),^80^ or copolymers.^81,82^ This approach disrupts the rigid crystalline structure of PEO while preserving a sufficiently low *T*_g_, ensuring that the polymer chains maintain the necessary mobility for efficient ion transport.

However, unlike block copolymers, which can be designed to form highly uniform ion-conducting channels, as shown in the previous section, polymer blends demand a detailed examination of their miscibility behavior.^77^ In blends, phase separation tends to occur over larger length scales, resulting in heterogeneous morphologies. This nonuniformity may lead to areas with elevated ionic resistance or mechanical weaknesses, potentially compromising both the performance and reliability of the battery. Standard characterization techniques used for studying SPE blends, such as DSC, FTIR, or XRD, only provide bulk information on thermal transitions and chemical interactions, failing to describe the miscibility behavior of blend components.^77^

In response, SANS has recently emerged as a powerful tool to fill this gap left by traditional SPEs characterization techniques. SANS not only enables probing of nanoscale morphology but also measures key parameters such as the effective Flory–Huggins interaction parameter (χ_eff_) that directly reflect the degree of miscibility between blend components. This insight is crucial, as both the polymer constituents and the inorganic salt influence the overall phase behavior, ultimately impacting the electrolyte’s performance. Thus, Gao et al. demonstrated how salt addition can promote a transition from macrophase segregation state to a single-phase system by effectively tuning the χ_eff_.^83^ SANS measurements revealed a distinct miscibility window where the incorporation of salt resulted in a homogeneous blend, even for polymers that are typically immiscible, such as PEO and poly­(1,3,6-trioxocane) (P­(2EO-MO)) (Figure 2C). Subsequent studies have expanded and confirmed these findings across a broader range of polymer systems, showing the potential of SANS for guiding the design of new blended SPEs.^84–87^

#### 4.1.3 Cross-Linking

Building on the previous section on polymer blending, researchers have advanced the design by incorporating monomers that can form covalent bonds between polymer chains.^88^ By introducing these reactive monomers, such as poly­(ethylene glycol) diacrylate (PEGDA) or polyethylene glycol methyl ether methacrylate (PEGMEMA) into the PEO polymer matrix,^89,90^ they enable a UV-induced cross-linking process that generates a robust three-dimensional network. This network not only disrupts the regular crystalline arrangement of the PEO chains but also significantly enhances the mechanical properties of the final SPE.

It is precisely through these enhanced mechanical properties that novel characterization techniques have provided fresh insights into the material’s behavior. Tensile stress–strain tests have traditionally been the primary method for evaluating the mechanical performance of SPEs. For instance, implementing cross-linking strategy on PEO matrix has improved the tensile strength of SPEs from approximately 10 MPa, with an elongation at break of around 5%, to about 45 MPa and 40%, respectively.^91^ However, while these tests provide essential static mechanical data, they fall short in elucidating the dynamic viscoelastic behavior of these materials under operational conditions.

In response, researchers have recently explored oscillatory rheology as a complementary technique for studying SPEs’ mechanical properties. By examining parameters such as the storage modulus (*G*′) and loss modulus (*G*″) across a range of frequencies and temperatures, Falco et al. demonstrated that cross-linked PEO shows a strongly elastic, gel-like behavior even at high temperatures (up to 130 °C), with *G*′ remaining nearly 1 order of magnitude higher than *G*″ over the entire frequency range, contrary to standard PEO (Figure 2D).^92^ This dynamic characterization not only confirms that the UV-induced cross-linking effectively stabilizes the network under thermal stress but also provides critical insights into its robustness during battery operation.

### 4.2 Novel Polymer Materials for Advanced Solid Electrolytes

Despite efforts in improving the capabilities of PEO-based electrolytes highlighted in previous sections, researchers are also exploring alternative polymer families that offer higher ionic conductivity and cationic transference number, enhanced thermal stability, improved mechanical properties, and broader electrochemical stability windows (Figure 3A).^93^

**Figure 3:**
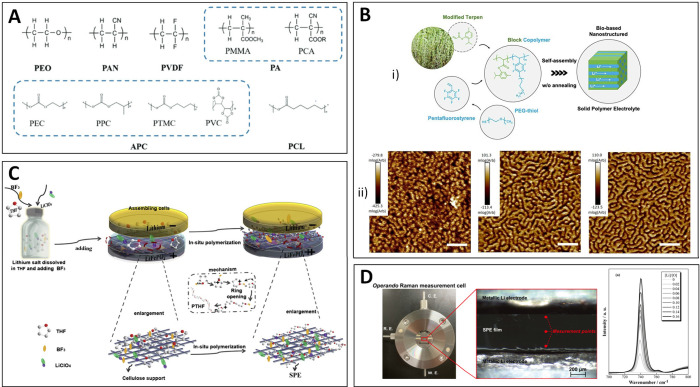
(A) Chemical structures of common polymer matrices for PSEs. Adapted with permission from ref 93. Copyright 2020 Wiley-VCH. (B) (i) Schematic of the preparation of nanostructured SPEs based on biomass-derived polymers and (ii) AFM topographical images of the nanostructures obtained after self-assembly. Adapted from ref 108. CC BY 4.0. (C) Schematic diagram of the in situ polymerization process of a PTHF SPE. Adapted with permission from ref 110. Copyright 2019 Elsevier. (D) Photograph of an operando Raman measurement cell and measurement points (left) and Raman spectra of salt concentration dependences obtained using the operando cell (right). Adapted from ref 111. Copyright 2023 American Chemical Society.

One prominent example, already mentioned in the introduction, is the use of PTHF, which is structurally similar to PEO (polyether) but contains fewer oxygen atoms in its backbone, resulting in a lower density of solvation sites.^27,94^ As a consequence, the coordination between oxygen and Li^+^ is significantly weakened compared to PEO. Polycarbonate-based electrolytes offer another promising alternative.^95^ Poly­(ethylene carbonate) (PEC) forms a looser coordination structure with Li^+^ compared to polyethers. In PEC, the carbonyl (C=O) groups interact with Li^+^ and the accompanying anions (such as bis­(fluorosulfonyl)­imide (FSI)) with sufficient strength to facilitate salt dissociation without overly impeding ion transport. FTIR and Raman spectroscopy studies have shown that the relatively weak coordination in PEC results in a higher ionic conductivity.^38^

Another well-known family of polymers, such as polyesters, has also shown promising results.^96^ Polyesters contain ester linkages, which offer a moderate interaction with Li^+^ ions.^97^ This moderate binding is important because if the interaction is too strong, it can limit ion mobility, and if it is too weak, the salt may not dissolve adequately. In many cases, the structure of polyesters has been tailored to reduce crystallinity, thereby increasing the amorphous regions in the polymer. These amorphous regions allow for easier movement of the polymer chains and create more pathways for Li^+^ transport. Polylactones, which are cyclic esters, exhibit similar advantages due to their flexible ring structures that support balanced ion coordination and transport.^98^

Polyacrylates (PAs), including poly­(methyl methacrylate) (PMMA) and polycyanoacrylate (PCA), offer additional benefits as they have functional ester groups with electron-donating abilities that promote salt dissociation.^99^ PMMA-based polymers are highly amorphous, which should be advantageous for ion movement, yet its brittleness and low ionic conductivity at room temperature have necessitated modifications such as blending with inorganic fillers or plasticizers (see next section for more details).^100^ PCA, with its strong binding ability due to nitrile groups, shows high electrochemical stability and is particularly promising for high-voltage batteries, although reliance on liquid plasticizers can compromise its mechanical properties.

In general, these weaker-coordination matrices still present challenges, especially with their oxidation stability above 4 V vs Li/Li^+^ (narrower than that of PEO), also their higher moisture sensitivity can raise limitations in processing and final mechanical stability of the SPE. For that reason, researchers have also been looking for novel synthetic protocols to further expand the range of accessible materials for SPEs. Recent works have shown the possibility of engineering the polymer chain in order to create ordered systems that facilitate ion conduction more effectively. For example, liquid crystal polymers can form organized structures that create directional channels for ion conduction. Thus, Kato produced a liquid crystal electrolyte by photopolymerizing polymethacrylate liquid crystal monomers directly in the reaction mixture.^101^ In this system, separate layers of flexible tetra­(ethylene oxide) segments and rigid aromatic cores form clear routes for LI^+^ movement. This arrangement resulted in an ionic conductivity of 10^–3^ S/cm along the aligned channels at room temperature.

Covalent-organic frameworks (COFs) represent another frontier in SPE design. These crystalline and porous materials provide well-defined channels that facilitate Li^+^ migration. In particular, imidazolate-containing ionic COFs have been synthesized as single-ion conductors. Zhang et al. reported that these COFs, with weak Li^+^-imidazolate interactions, achieve ionic conductivities up to 7.2 × 10^–3^ S/cm at room temperature.^102^ By incorporating electron-withdrawing substituents, the ion-pair interactions are further weakened, thus enhancing the conduction properties. Additionally, polyelectrolyte COFs, where flexible oligo­(ethylene oxide) chains are integrated into the framework, have been shown to improve Li^+^ mobility by providing a favorable polyelectrolyte interface.^103^ Practical uses, however, are still limited mainly by poor contact between microcrystalline COF powders and electrodes; the resulting interfacial voids promote lithium-dendrite nucleation during cycling, compromising both performance and cell life.

Porous aromatic frameworks (PAFs) also offer promising avenues for SPEs. Zou et al. introduced a porous polymer electrolyte where LiPF_6_ is adsorbed within the aromatic channels of a porous aromatic framework (PAF-1).^104^ The design leverages high binding energy between Li^+^ and the aromatic rings, facilitating fast Li^+^ transport through loosely bound ions occupying the remaining void space. The resulting SPE exhibits an ionic conductivity of 4.0 × 10^–4^ S/cm at room temperature and maintains high electrochemical stability, however, the powdery nature of PAFs makes it challenging to cast defect-free membranes and achieve good electrode contact, issues that still need to be resolved for practical use.

Covalent adaptable networks (CANS) are emerging as key components in SPEs for lithium batteries due to their dynamic covalent bonding, which allows for reprocessability, recyclability, and self-healing. These materials combine the mechanical robustness and thermal stability of thermosets with the malleability of thermoplastics, enabling flexible and durable electrolytes that can adapt to mechanical stress, suppress lithium dendrite growth, and maintain stable interfaces. Additionally, their ability to undergo bond exchange reactions facilitates the restoration of conductivity and mechanical properties after damage, offering a sustainable solution for safer and longer-lasting solid-state batteries.^105–107^

In parallel with these synthetic advancements, researchers are increasingly turning to eco-friendly, bio-based electrolytes. For example, terpene-based block copolymer, derived from biomass, has recently been introduced as a new family of SPEs (Figure 3B).^108^ Moreover, these new polymer systems have also shown their ability to generate well-defined self-assembled structures, and provide conductivity values comparable to other nanostructured petroleum-based SPEs (10^–4^ to 10^–3^ S/cm). Moving such materials toward commercial cells, however, still raises several practical questions. For instance, the ester/ether linkages that make terpenes and other bio-based polymers biodegradable are also highly hygroscopic, making it difficult to fully remove absorbed water. This persistent moisture can accelerate hydrolytic degradation, leading to faster cell aging and compromising the mechanical integrity of the SPE.^98^ End-of-life handling is equally challenging: Residual salts and fragmented electrode materials complicate mechanical recycling, as they can contaminate recovered polymers or metals, while targeted bond-cleavage processes remain limited to laboratory demonstrations.^109^

Advances are happening not only in the polymerization reactions but also in their practical implementation. One of the most interesting developments is the in situ polymerization. In this method, monomers are injected directly into the battery cell and then polymerization is initiated by introducing a suitable initiator (Figure 3C).^110^ This solvent-free process forms an SPE directly on the electrode surface, ensuring excellent interfacial contact. SPEs produced in this manner exhibit high ionic conductivities (greater than 1.0 mS/cm at room temperature) and low interfacial resistance, while also maintaining high Coulombic efficiency over many cycles. The development of the in situ polymerization technique has been closely accompanied by intensive work on in situ chemical characterization methodologies. Thus, spectroscopy techniques such as FTIR or Raman, and solid-state NMR have been adapted in order to be able to monitor and follow the processes happening in the battery cell (Figure 3D).^111^

### 4.3 Composite SPEs: Engineering Additives for Enhanced Ionic Conductivity

Another alternative methodology for creating advanced SPEs is through solid composite electrolytes (SCEs). Thus, by integrating additives such as plasticizers or inorganic fillers into the polymer matrix, it is possible to enhance ionic conductivity, bolster mechanical strength, and expand the electrochemical stability window, thereby overcoming many limitations of single-component SPEs.^112^

Plasticizers are frequently added to SPEs to reduce crystallinity and increase free volume, thereby facilitating ion transport. In many systems, the addition of small-molecule plasticizers disrupts the rigid packing of polymer chains, lowering the *T*_g_ and enhancing chain mobility.^113^ For example, in polyacrylonitrile (PAN)-based electrolytes, plasticizers such as propylene carbonate (PC), succinonitrile (SN), and polyethylene glycol dimethyl ether (PEGDME) can effectively interrupt the strong coordination between Li^+^ and the nitrile groups.^114^ Although pristine PAN/LiClO_4_ systems exhibit extremely low ionic conductivity (∼10^–7^ S/cm), the incorporation of these plasticizers has been shown to boost conductivity to around 10^–6^ S/cm by promoting salt dissociation, all while maintaining the high electrochemical stability and wide voltage window inherent to PAN-based systems.

In addition to plasticizers, inorganic fillers play a crucial role in engineering composite electrolytes. They not only provide additional ion transport pathways via Lewis acid–base interactions but also fundamentally alter the polymer’s crystallinity. Inert fillers such as ultrafine SiO_2_ particles, Al_2_O_3_, and TiO_2_ reduce the crystallinity of the polymer matrix, thereby promoting an amorphous phase that facilitates Li^+^ migration.^20^ Recent operando grazing-incidence small- and wide-angle X-ray scattering (GISAXS and GIWAXS) studies have revolutionized the characterization of these inorganic filler-based electrolytes under real operating conditions (Figure 4).^115^ These advanced techniques enable real-time monitoring of lithium plating and delithiation processes in a Li–Cu cell, directly linking electrochemical reactions to structural evolution. During cycling, the operando GIWAXS data reveal a marked decrease in the intensity and an increase in the broadening of the PEO-Li coordination peak, clear indicators of reduced crystallinity in the PEO matrix. Simultaneously, GISAXS measurements document changes in domain size and interdomain distances, parameters that critically influence the formation of ion transport channels. Together, these insights confirm that inorganic fillers not only induce a more amorphous, disordered structure but also enhance ion transport, ultimately leading to improved battery performance.

**Figure 4:**
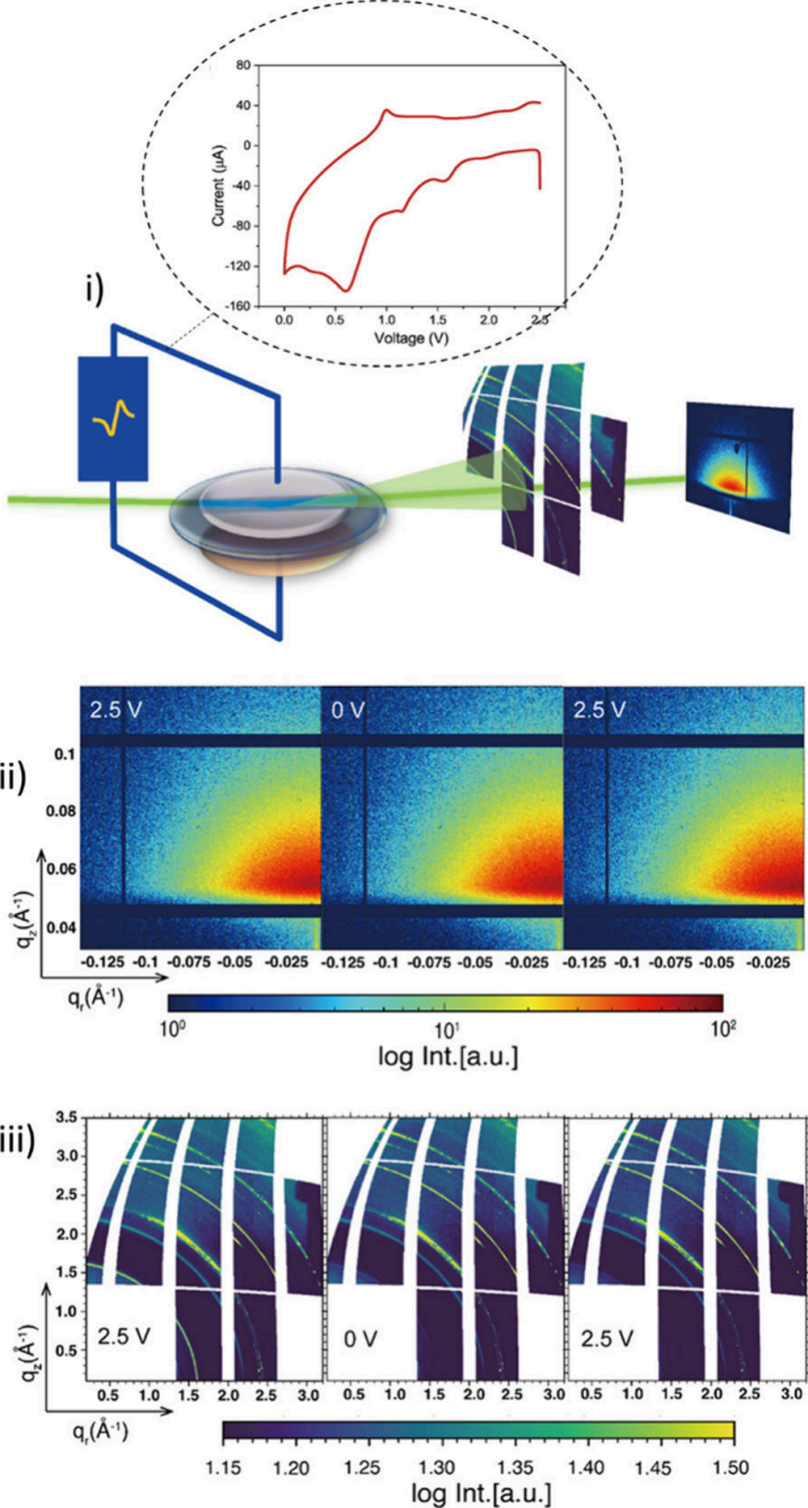
(i) Schematic representation of the synchrotron-radiation-based GISAXS/GIWAXS operando experiment under cycling conditions. (ii, iii) Examples of the (ii) 2D GISAXS and (iii) 2D GIWAXS data obtained during the experiment. Adapted from ref 115. CC BY 4.0.

In addition to these inert fillers, inorganic additives can also be active, directly participating in LI^+^ conduction. For example, garnet-type ceramics such as Li_7_La_3_Zr_2_O_12_ (LLZO),^116^ LiAlO_2_,^117^ and their derivatives,^118^ have emerged as prominent active fillers due to their high ionic conductivity and chemical stability. When embedded in a polymer matrix, LLZO not only provides additional fast-ion conduction channels but also helps form a robust solid electrolyte interphase (SEI) on the lithium metal anode. Composite electrolytes incorporating LLZO nanowires into a PAN-LiClO_4_ matrix have demonstrated enhanced conductivity (up to 2.4 × 10^–4^ S/cm at room temperature) as Li^+^ preferentially migrate through the modified interfacial regions between LLZO and the polymer.^119^ Moreover, strategies such as coating LLZO with surfactants such as cetyltrimethylammonium bromide (CTAB) have been employed to stabilize the fillers, avoiding their aggregation and improving their interfacial contact at the SPE interface.^120^

## 5 Integrating Innovation and Characterization: A Roadmap

In previous sections, we showed that major advancements in SPE materials depend on equally robust progress in their characterization. Information from computational models and experiments can help guide polymer design. Complementary, detailed chemical analyses reveal key insights that help us predict and refine our understanding of a material’s final properties. This combined approach creates a beneficial cycle where improved synthesis and enhanced characterization mutually support each other, advancing the field as a whole.

### 5.1 From Static to Dynamic

In previous sections, we have seen how dynamic processes are influenced by structural changes and how advanced structural characterization techniques have deepened our understanding of the link between these changes and overall battery performance. However, there is still much to learn about the dynamic processes occurring inside SPEs. QENS has been instrumental in revealing dynamic processes in many fields, from probing protein motions in biological systems to studying molecular transport in complex fluids,^121^ and it is now beginning to provide similar insights into SPEs.^122^

In SPEs, QENS has emerged as a powerful tool to probe the segmental motion of polymer chains, providing a direct measure of chain dynamics that are closely linked to ion transport. Researchers have observed that the presence of lithium salts is correlated with a slowdown in these segmental motions, a phenomenon that has enabled the definition of a “monomeric friction coefficient”.^123^ This coefficient, which increases with salt concentration, serves as a quantitative indicator of how the polymer matrix’s local dynamics are affected by ion coordination. Likewise, similar QENS studies have shown that SPEs’ segmental dynamics are very sensitive to the material’s thermal history, as thermal treatment influences the degree to which the amorphous regions are confined within the surrounding crystalline areas. For example, a (PEO)_16_LiTf sample equilibrated at room temperature for a week exhibits a 6-fold longer structural relaxation time compared to a sample that was just cooled down, resulting in a 3-fold lower ionic conductivity.^124^

Beyond polymer dynamics, QENS has also been applied to study ion transport itself. Thus, recent measurements have indicated that lithium solvation dynamics occur on a nanosecond time scale, much slower than previously assumed in analogous liquid systems.^125^ This slower dynamic suggests that LI^+^ may form transient cross-links with multiple polymer chains. The formation of these cross-links reinforces the polymer network, enhancing its mechanical strength and thermal stability, having important implications for the design of electrolytes that combine high safety with fast charging capabilities.

Despite these advances, the application of QENS to SPEs is still in its early stages. Looking ahead, a central challenge will be to consolidate multiscale observations into a unified framework capable of predicting and optimizing battery performance. This roadmap calls for advanced characterization protocols that marry in situ and operando neutron scattering techniques with atomistic simulations. By capturing experimental feedback on both polymer segmental dynamics and lithium solvation processes, researchers can directly correlate structural features with dynamic behavior under realistic operating conditions. At the same time, the development of refined computational models is essential to reconcile the wide range of time scales, from rapid solvation to slower collective polymer motions, which govern ion transport.

### 5.2 From Isolated Probes to Integrated Characterization

As previously introduced, SPE research has routinely paired chemical characterization techniques (e.g., NMR, FT-IR, elemental analysis) with physical tests such as TGA, DSC, or rheology to cross-validate composition, stability, and conductivity. While this “mix-and-match” strategy remains indispensable, the past decade has witnessed a decisive shift toward simultaneous or sequential integration of advanced X-ray and neutron characterization methodologies inside large-scale facilities, allowing researchers to obtain data from a single specimen across complementary time- and length-scales without removing it from a controlled environment. Thus, moving beyond the traditional “one sample, one technique” paradigm offers two decisive benefits. First, it unifies structure and dynamics: ultrafast motions captured by neutron probes (QENS, inelastic/time-of-flight) can be interpreted in the light of nano- to mesoscale morphologies revealed by X-ray or neutron scattering. Second, it removes sample-to-sample variability because every dataset is recorded on the same specimen under identical temperature, hydration, or electrochemical bias. This multimodal approach was recently applied to mapping hydroxide-ion transport in a single anion-exchange membrane.^126^ Thus, before and after each QENS measurement, the sample underwent small- and wide-angle X-ray scattering (SAXS/WAXS) and small-angle neutron scattering (SANS). The scattering data quantified 1–10 nm ion-channel widths and their swelling with hydration, while QENS yielded activation energies and jump lengths for OH^–^ diffusion. The overlap of these datasets provides a direct structure-dynamics-conductivity map that guides rational membrane design. Such integrated measurements will be invaluable for studying nanostructured SPEs, like the ones discussed in Sections 4.1 and 4.2.

### 5.3 From Data Generation to Predictive Modeling

Another promising direction is to combine detailed chemical characterization with computational simulations to create prediction models based on machine learning (ML). Similar approaches have already been applied to fields that require high synthetic efforts, such as colloidal chemistry and pharmaceuticals, but their application to predicting novel SPE formulations or modifications to polymer chains is still starting to be explored.

The rationale behind this approach is straightforward. Datasets derived from advanced measurements, such as high-resolution spectroscopy, diffraction, and microscopy, along with simulation outputs, obtained using simulation suited such as LAMMPS or GROMACS, capturing parameters like molecular structure, phase segregation, ion coordination, and polymer segmental dynamics, provide the essential groundwork to understand how subtle changes in composition, polymer chain architecture, and structure influence the performance of SPEs. Open-source libraries such as Matminer or DScribe can be used to convert both experimental and simulated outputs into machine-readable feature vectors. With these robust datasets in hand, ML models, such as polymer-specific platforms like Polymer Genome or the Materials Project API, can help researchers identify correlations between them and build predictive models that guide synthesis.

The potential is already clear. Recent work combined a Matminer-style descriptor set with ab initio simulation data and trained a temperature-aware neural network (implemented in PyTorch) to predict ionic conductivity across hundreds of published SPE compositions.^42^ The resulting model screens thousands of hypothetical formulations in silico, narrowing experimental efforts to the most promising candidates and illustrating how an integrated software stack can accelerate SPE discovery.

### 5.4 From Waste to Product

Next-generation SPEs must deliver not only superior performance but also meet the demands of scalability and sustainability. Some of the most promising approaches to achieve these goals have already been discussed in this work, including in situ solvent-free processing. Techniques such as UV or thermal curing of cross-linkable polymers, like poly­(ethylene glycol) diacrylate, enable roll-to-roll manufacturing.^127^ Recent advances in solvent-free PTHF-based SPEs have demonstrated competitive conductivities of 10^–5^ S/cm and exceptional electrochemical stability.^128^ This, combined with their flexibility and free-standing nature, highlights their potential for high-performance production without the disadvantages associated with volatile organic compounds.

The incorporation of bio-based or biodegradable polymers represents another key strategy. By using polyesters such as poly­(caprolactone) (PCL) and poly­(ethylene succinate), manufacturers can design systems that offer both competitive performance, around 10^–4^ S/cm conductivity, and full biodegradability. As previously introduced, concerns about their tendency to retain trace moisture due to hygroscopic functional groups remain. Several routes are being explored to overcome these drawbacks, such as copolymerizing the PCL and polyester with more robust blocks, preserving chain mobility while also improving mechanical and electrochemical endurance.^129^ Finally, blending these polymers with bioderived plasticizers, such as lignin-based additives, not only enhances the ionic conductivity but also contributes to a circular economy by enabling recyclability and reducing environmental impact.^130^

## 6 Conclusion and Outlook

SPEs today occupy a similar position to LI^+^ cells three decades ago: numerous proofs of concept exist and pilot lines are emerging, yet none of the current formulations simultaneously meet the stringent industrial requirements for ionic conductivity, interfacial durability, mechanical robustness, electrochemical stability, scalable cost, and end-of-life recyclability. In this Perspective, we have traced how advances in synthetic design, copolymerization, blending, cross-linking, and novel backbone chemistries, must be anchored to equally rigorous, characterization-driven understanding of polymer dynamics, microstructure, and interfacial chemistry. Only by integrating these insights can SPEs cross the final line between lab-scale performance and factory-ready reliability. During the preparation of this work, some grand challenges have arisen, which we believe must be overcome in order to bring SPEs into commercial reality.

Thus, in a world increasingly focused on sustainability, the battery sector cannot be exempt. SPEs already eliminate leakage of hazardous liquids, but relying on petroleum-based polymers merely swaps one environmental burden for another. Transitioning to bio-based SPEs, such as terpene-derived block copolymers and biodegradable polyesters, must therefore be a priority. However, these materials introduce new challenges, particularly their high moisture uptake due to hygroscopic functional groups. This can lead to persistent water retention, accelerating hydrolytic degradation, and promoting interfacial instability, as discussed in previous sections. Quartz-crystal microbalance with dissipation monitoring (QCM-D) provides a direct, in situ method to observe mass changes and viscoelastic responses as bio-based polymers retain residual water and begin to undergo hydrolysis; by coupling QCM-D with electrochemical measurements (E-QCM-D), one can simultaneously track ionic currents and mechanical softening. These insights are essential not only for understanding degradation mechanisms but also for guiding the development of more robust polymer chemistries, such as in situ polymerization strategies or depolymerization routes that enable closed-loop recycling without compromising battery performance.

Also, in situ polymerization must become mainstream, not only to eliminate solvent use and enable roll-to-roll coating, but also to lock in high-voltage stability. By injecting a UV or thermally curable monomer/initiator mix directly into the cell and triggering polymer growth on the electrode surfaces, the SPE forms without residual solvents or interfacial voids that normally decompose at high voltages. In this sense, monomers with oxidation-resistant groups (e.g., aromatic methacrylates or sulfone-linked diacrylates) should be paired with nanoscale interlayers that improve wetting, while operando FTIR and QCM-D monitoring can be useful to follow the cure and interface integrity in real time.

Additionally, the long-standing trade-off between soft, ion-conductive matrices and the mechanical robustness needed to suppress dendrites must be shattered by smarter polymer architectures, not just conventional block copolymers. CANs offer a powerful solution: their dynamic bonds enable self-healing and tunable stiffness (as shown in Section 4), while retaining the malleability required for ion transport. By designing CAN-based block copolymers in which one segment is a soft, amorphous ionophilic block (e.g., PEO, PTHF, or polycarbonate) and the other carries reversible linkages (boronic esters, imines, or disulfides), we can lock in continuous ion channels within a mechanically resilient skeleton.

Finally, as seen in Section 2, industry is progressing quickly with upscaling lithium-based solid-state batteries and beginning to move beyond lithium, exploring silicon-based anodes, aluminum-graphite cells, and multivalent metals. This diversification makes it vital that our characterization toolbox remains versatile. Neutron scattering, with its unique ability to tune contrast (for example, by selectively deuterating the polymer matrix), offers an unequaled opportunity to isolate signals from polymer, salt, and electrode interfaces, even in complex, multimetal systems. Seizing this capability will ensure that the next generation of SPEs can be confidently tailored for a broad range of battery chemistries. This integrated approach will accelerate scale-up and deliver the robust, high-performance, and environmentally responsible electrolytes that next-generation batteries urgently require.
